# Pellagra and Beri-Beri

**Published:** 1915-05

**Authors:** L. B. Bibb


### EDITORIAL DEPARTMENT.

MRS. F. E. DANIEL, Publisher and Managing Editor.
DR. R. H. L. BIBB, Associate Editor.

CONTRIBUTING EDITORS:

Dr. H. Sheridan Baketel, New York, editor *Medical Times;* Dr. M. H. Boerner, Austin, Texas; Dr. G. Henri Bogart, Paris, Ill.; Dr. M. M. Carrick, Dallas, Texas; Dr. Edward H. Carey, Dean Medical Department, Baylor University, Dallas, Texas; Dr. W. L. Crosthwaite, Waco, Texas; Dr. T. D. Crothers, Hartford, Conn.; Dr. H. W. Cummings, Hearne, Texas; Dr. Oscar Dowling, New Orleans, President Louisiana State Board of Health; Havelock Ellis, M. D., London, Eng.; Dr. May Agnes Hopkins, Dallas, Texas; Dr. A. W. Fly, Galveston, Texas; Dr. G. B. Foscue, Waco, Texas; Dr. E. S. Goodhue, Holualoa, Kona, Hawaii; Dr. M. B. Grace, Seguin, Texas; Dr. Joe Gilbert, Austin, Texas; Dr. Charles H. Hughes, St. Louis, editor *Alienist and Neurologist;* Dr. James G. Kiernan, Chicago; Dr. George H. Lee, Galveston, Texas; Dr. John T. Moore, Houston, Texas; Dr. Frank Paschal, San Antonio, Texas; Dr. John Preston, Austin, Texas, Superintendent State Lunatic Asylum; Dr. G. Wilse Robinson, Kansas City, Mo.; Dr. Bacon Saunders, Fort Worth, Texas; Dr. Allen J. Smith, Philadelphia, Medical Department, University of Pennsylvania; Dr. Z. T. Scott, Austin, Texas; Dr. Ralph Steiner, Austin, Texas, President Texas State Board of Health; Dr. Walter S. Sutton, Kansas City, Mo.; Dr. A. E. Sweatland, Nacogdoches, Texas; Dr. John S. Turner, Dallas, Texas; Dr. Charles Venable, San Antonio, Texas.

### Pellagra and Beri-Beri.

WRITTEN FOR TEXAS MEDICAL JOURNAL BY L. B. BIBB, M. D.

The solution of the beri-beri problem is an accomplished fact. That the pericarp of rice contains a "neuritis preventing principle," often called vitamine, is absolutely proved by many tests, confirmed and reconfirmed by a dozen investigators.

From the vantage point thus gained a new insight regarding pellagra is given to the profession. Pellagra closely resembles beri-beri epidemiologically; both diseases occur in institutions, labor camps, jails, and among the poor. A positively contagious or infectious quality has never been proved in either disease. Both diseases affect the nervous system and other parts also.

Experiments consisting of feeding animals on a diet of artificial mixtures of pure protein, fats and carbohydrates indicate that not only does the body need protein of certain quality, but also it must have certain lipoid constituents which cannot be synthesized in the body. These experiments may explain the etiology of beri-beri and of pellagra. A diet may contain all the protein needed yet the protein may not yield all the varieties of amino acids needed; and it may be deficient in cholesterin and other lipoids. Such a diet in animals leads to stoppage of growth and even death. Might it not lead to pellagra in man? When thought of in this light, pellagra fits it snugly, and can be satisfactorily explained.

After the chemists have determined exactly which amino acids can be derived from each protein, and in what proportion; and after the physiologists have determined which amino acids and in which combinations are essential to growth, health and life, then a clear understanding of diet will be possible.

